# A Novel Clinically Prognostic Stratification Based on Prognostic Nutritional Index Status and Histological Grade in Patients With Gallbladder Cancer After Radical Surgery

**DOI:** 10.3389/fnut.2022.850971

**Published:** 2022-05-04

**Authors:** Peng Cao, Haijie Hong, Zijian Yu, Guodong Chen, Shuo Qi

**Affiliations:** ^1^Department of Hepatopancreatobiliary Surgery, The First Affiliated Hospital, Hengyang Medical School, University of South China, Hengyang, China; ^2^Department of Hepatobiliary Surgery and Fujian Institute of Hepatobiliary Surgery, Fujian Medical University Union Hospital, Fujian Medical University Cancer Center, Fuzhou, China

**Keywords:** gallbladder carcinoma, prognosis, prognostic nutrition index, histological grade, radical resection

## Abstract

**Purpose:**

Gallbladder carcinoma (GBC) is the most common malignancy of the biliary tract, with a 5-year survival rate of 5%. The prognostic models to predict the prognosis of patients with GBC remain controversial. Therefore, to construct a prognosis prediction of GBC, a retrospective cohort study was carried out to investigate the prognostic nutritional index and histological grade in the long-term outcome of patients with GBC after radical surgery (RS).

**Methods:**

A retrospective study of a total of 198 patients with GBC who underwent surgical treatment were enrolled. The hematological indicators, imageological data, and perioperative clinical data were acquired for statistical analysis and poor prognosis model construction.

**Results:**

Prognostic nutrition index (PNI) < 45.88, maximum tumor diameter (MTD) > 2.24 cm, and jaundice (JD) were all associated with a poor prognosis in multivariate logistic regression analysis. The prognosis prediction model was based on the three risk factors, which indicated a superior predictive ability in the primary cohort [area under the curve (AUC) = 0.951] and validation cohort (AUC = 0.888). In multivariate Cox regression analysis, poorly differentiation (PD) was associated with poor 3-year survival. In addition, Kaplan–Meier (KM) survival analysis suggested that GBC patients with high-risk scores and PD had a better prognosis after RS (*p* < 0.05), but there was no significant difference in prognosis for patients with non-poorly differentiation (NPD) or low-risk scores after RS (*p* > 0.05).

**Conclusion:**

Our prediction model for GBC patients with prognosis evaluation is accurate and effective. For patients with PD and high-risk scores, RS is highly recommended; a simple cholecystectomy can also be considered for acceptance for patients with NPD or low-risk score. The significant findings provide a new therapeutic strategy for the clinical treatment of GBC.

## Introduction

Gallbladder carcinoma (GBC) is the most common malignancy of the biliary tract (1). The surveillance, epidemiology, and end results program estimated that the incidence of GBC was 2.5 per 100,000 persons (2, 3). GBC is difficult to be diagnosed at an early stage due to the symptomless nature. When an accurate diagnosis is made, radical cure often cannot be implemented due to the direct invasion into adjacent structures, such as the hepatic artery or portal vein, as well as metastasis *via* the lymphatic, perineural, and hematogenous routes (4–6). The median overall survival (OS) for GBC was about 6 months, with a 5-year survival rate of 5% (7, 8). Therefore, it is important to improve the early diagnostic rate of patients with GBC and evaluate their prognosis perioperative-operation.

Although various scoring systems are used in clinical practice, the preoperative prognostic models to predict the prognosis of patients with GBC remain controversial (9–11). These models were based on a number of hematological and clinical indicators, such as prognostic nutrition index (PNI), the diameter of tumor, jaundice, and TNM stage (12–15). Numerous clinical pieces of evidence have shown that the PNI was associated with prognosis in patients with digestive tract malignancies, such as hepatocellular carcinoma, gastric carcinoma, pancreatic carcinoma, and colorectal carcinoma (16–19). Moreover, several studies have investigated the relationship between histological grade and prognosis of endometrial and breast cancer (20, 21). However, the PNI and histological grade have not yet been determined in the prediction of prognosis in patients with GBC. Therefore, to construct a poor prognosis prediction of GBC to guide its treatment, we conducted a retrospective cohort study of patients with GBC to investigate the PNI and histological grade indicators in the long-term outcome.

## Patients and Methods

### Study Population

We conducted a retrospective study on a total of 198 patients with GBC who underwent surgery in the Department of Hepatobiliary Surgery of Fujian Medical University Union Hospital between January 2008 and December 2017. The study was carried out in accordance with the protocol approved by the Ethics Committee of the Medical Faculty of Fujian Medical University, according to the Declaration of Helsinki.

All the patients included in the present study fit the following criteria: (1) patients with GBC underwent surgery [radical surgery (RS) or non-radical surgery (NRS)]; and (2) neither radiotherapy nor chemotherapy was administered prior to or posterior to the surgery. The histological diagnosis of the tumors was based on the criteria of the World Health Organization (WHO), and the TNM stage was determined according to the American Joint Committee on Cancer (AJCC, 7th). The patients with the following characteristics were excluded: (1) the medical history, operation records, and auxiliary examination are incomplete; (2) death occurred during and after the operation; (3) patients are not willing to cooperate with the investigation during the follow-up; and (4) patients with coexisting or previous cancers. Based on the hospital database, the following data were collected for each patient, such as age, gender, T stage, and other miscellaneous clinical characteristics.

### Analysis of Indicators

The prognostic nutrition index was calculated from the baseline clinical peripheral lymphocyte count (PLC) (*10^9^/L) and serum albumin (ALB) (g/L) within 1 week before surgery as follows (22, 23): PNI = ALB (g/L) + 5 × PLC (*10^9^/L). Jaundice was defined as yellow staining of the sclera of the patients (serum bilirubin > 34 mmol/L). Patients with postoperative recurrence/metastasis/death time less than 36-months were considered to have poor prognosis. The obtained hematological index and imageological index were established with a receiver operating characteristic (ROC) curve of poor prognosis. The cut-off values of these variables were obtained according to the best Youden index when these areas under the curve (AUC) were more than 0.6, and then classified into two categories; the classification criteria for each potential index were determined through the literature when the AUC was less than 0.6.

### Follow-Up Assessments

All of the patients were followed by telephone interviews or outpatient reviews. The duration of follow-up was defined as the time between the date of operation and the last follow-up before the data were analyzed, or the date of death. The patients received follow-ups until December 2020. The patients were followed up every 3 months during the first postoperative years, and every 6 months for the next 2 years. Physical examination, peripheral blood tumor marker measurements (Ca199 and CEA), and pectoral and abdominal computed tomography (CT) or magnetic resonance imaging (MRI) were performed during the follow-up period. The median follow-up duration was 17.5 months (range 1–36 months). The follow-up rate of this study was 87.9%.

### Statistical Analysis

The statistical analyses were performed by standard SPSS (version 25.0, IBM, Armonk, NY, United States). The categorical variables were presented as numeric values and percentages, and the continuous variables with normal distributions were presented as means and standard deviations (mean ± SDs). An independent *t*-test was used to compare the groups of continuous, normally distributed variables. Pearson’s χ^2^ test was used to determine the significance of the differences for the dichotomous variables. Univariate analysis and multivariate analyses with the logistic/Cox regression proportional hazard model were performed to evaluate the prognostic factors. OS survival and overall recurrence were defined as the time from operation until death or censoring, which were calculated by the Kaplan–Meier (KM) analysis, and the difference in groups was assessed by the log-rank test. All values of ps were two-sided, with statistical significance set at *p* less than 0.05.

## Results

### Study Design

**SCHEME 1:**
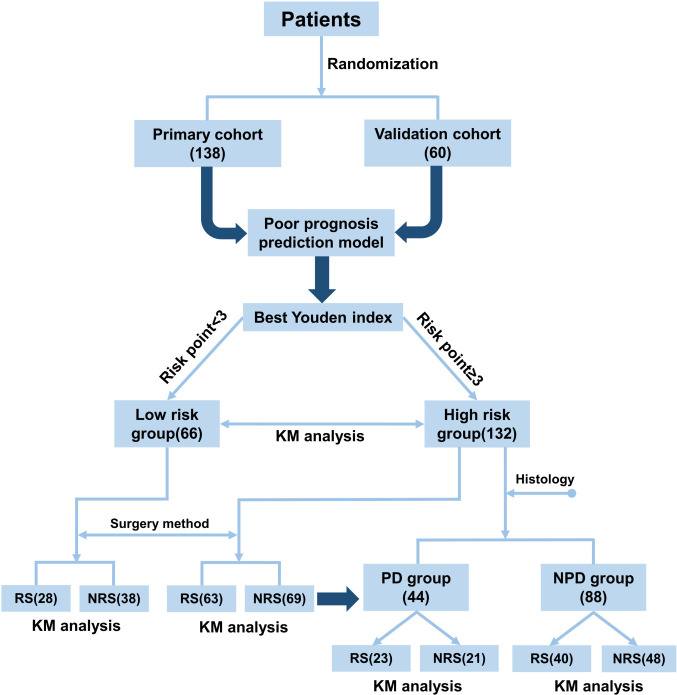
Flowchart of manuscript design.

The flowchart of the manuscript design is presented in Scheme 1. In this study, a total of 198 patients with GBC were enrolled and randomly assigned to the primary cohort (*n* = 138) and validation cohort (*n* = 60) in a ratio of 7:3. A poor prognosis prediction model was built and validated based on the clinical and laboratory indicators. According to the best Youden index, all patients were divided into high-risk and low-risk groups based on an optimal cut-off value. Subsequently, survival analyses were compared between high-risk and low-risk groups and their subgroups.

### Patients Characteristics

The baseline hematological, imageological, and pathological characteristics in the primary cohort and validation cohort are shown in Table 1. Among the two cohorts, the factors, such as PNI, age, gender, maximum tumor diameter (MTD), CEA, CA199, lymph node metastasis (LNM), TNM staging (I–IV grade), histological grading (poorly differentiation-PD and non-poorly differentiation-NPD), histological type, jaundice, cholelithiasis, tumor location, liver invasion, choledoch invasion, diabetes, hypertension, smoking, and poor prognosis between the two groups, showed no significant difference (*p* > 0.05).

**TABLE 1:** Baseline characteristics of study patients.

Variable	Primary cohort	Validation cohort	*P*-value
PNI	46.36 ± 5.92	45.67 ± 7.46	0.487
Age (years)	57.37 ± 10.17	57.02 ± 11.68	0.831
**Gender**			
Female	103(74.64)	45(75.00)	0.957
Male	35(25.36)	15(25.00)	
MTD (cm)	2.41 ± 0.39	2.21 ± 0.45	0.905
CEA (ng/mL)	3.03 ± 0.52	3.15 ± 0.53	0.140
CA199 (kU/L)	158.42 ± 234.06	189.25 ± 227.58	0.391
**LNM**			
Positive	42(30.43)	19(31.67)	0.863
Negative	96(69.56)	41(68.33)	
**TNM staging**			0.536
I	21(15.22)	5(8.33)	
II	47(34.06)	20(33.33)	
III	49(35.50)	23(38.34)	
IV	21(15.22)	12(20.00)	
**Histological grading**			
PD	43(31.16)	21(35.00)	0.622
NPD	95(68.84)	39(65.00)	
**Histological type**			
Adenocarcinoma	126(91.30)	58(96.67)	0.176
Other types	12(8.70)	2(3.33)	
**Jaundice**			
Present	19(13.77)	11(18.33)	0.410
Absent	119(86.23)	49(81.67)	
**Cholelithiasis**			
Present	40(28.99)	21(35.00)	0.400
Absent	98(71.01)	39(65.00)	
**Tumor location**			
Neck	69(50.00)	26(43.33)	0.388
Others	69(50.00)	34(56.67)	
**Liver Invasion**			
Present	97(70.29)	39(65.00)	0.461
Absent	41(29.71)	21(35.00)	
**Choledoch Invasion**			
Present	64(46.38)	27(45.00)	0.858
Absent	74(53.62)	33(55.00)	
**Diabetes**			
Present	30(21.74)	15(25.00)	0.615
Absent	108(78.26)	45(75.00)	
**Hypertension**			
Present	25(18.12)	15(25.00)	0.268
Absent	113(81.88)	45(75.00)	
**Smoking**			
Present	27(19.57)	13(21.67)	0.735
Absent	111(80.43)	47(78.33)	
**Poor prognosis**			
Present	96(69.57)	41(68.33)	0.868
Absent	43(30.43)	19(31.67)	

### Model Built and Validation

**FIGURE 1:**
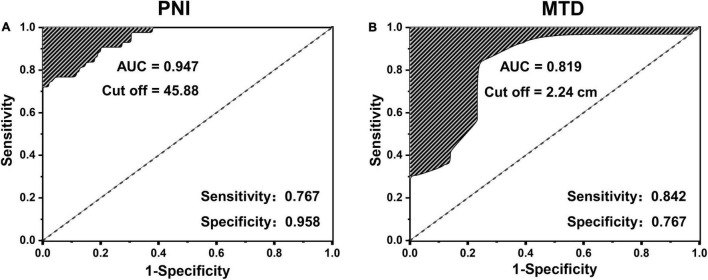
The receiver operating characteristic (ROC) curve of poor prognosis established with the candidate factors of panel **(A)** prognostic nutrition index (PNI) and **(B)** maximum tumor diameter (MTD).

The ROC curve of poor prognosis established with the candidate factors of PNI and MTD is shown in Figure 1. The AUC of PNI was 0.947, which was more than 0.6, and the cut-off value was 45.88. The AUC of maximum tumor diameter (MTD) was 0.819 and the cut-off value was 2.24 cm. Therefore, these two values were used to classify the classification variables in the next regression analysis.

The eighteen candidate risk factors related to poor prognosis were screened by univariate logistic regression analysis in the primary cohort which are shown in Table 2, and the positive results were, respectively, PNI < 45.88 (odds ratio [*OR*]: 3.508, *p* < 0.001), MTD > 2.24 cm (MTD, *OR*: 1.116, *p* < 0.001), PD (*OR*: 5.897, *p* = 0.027), Jaundice (JD, *OR*: 3.140, *p* = 0.006), liver invasion (*OR*: 2.302, *p* = 0.030). After multivariate analysis, the positive results were, respectively,: PNI < 45.88 (*OR*: 3.269, *p* < 0.001), MTD > 2.24 cm (*OR*: 0.075, *p* = 0.002), JD (*OR*: 3.059, *p* = 0.021). The multivariable analysis of these risk factors of poor prognosis and measurement of the risk scores are presented in Table 3. The prognosis prediction model was obtained by adding the total number of points scored in each of the three independent risk factors. The model was: poor prognosis risk = 4 × PNI + 2 × MTD + 2 × JD. The highest score was 8, and the lowest score was 0.

**TABLE 2:** Univariable and multivariate regression analyses of factors for the presence of poor prognosis in the primary cohort.

	Univariate analysis			Multivariate analysis		
Variable	OR	95%CI	*P*-value	OR	95%CI	*P*-value
PNI < 45.88	3.508	2.203–5.583	<0.001	3.269	2.026–5.043	<0.001
Gender	0.825	0.365–1.865	0.644			
Age < 60	1.121	0.858–1.856	0.785			
LN metastasis	1.676	0.734–3.827	0.220			
TNM staging	0.561	0.233–1.411	0.056			
MTD > 2.24 cm	1.116	1.049–2.273	<0.001	0.075	1.015–2.374	0.002
CEA < 5 ng/mL	0.768	0.458–1.569	0.485			
CA199 < 40 kU/L	0.895	0.396–1.636	0.652			
PD	5.897	1.225–8.378	0.027	3.288	0.133–8.514	0.467
Pathology	1.115	0.317–3.925	0.865			
Jaundice	3.140	1.839–6.033	0.006	3.059	1.494–5.751	0.021
Cholelithiasis	1.080	0.486–2.401	0.851			
Tumor location	1.610	0.778–3.333	0.200			
Liver Invasion	2.302	1.082–4.894	0.030	1.741	0.454–6.682	0.419
Choledoch Invasion	1.643	0.644–4.188	0.298			
Diabetes	2.027	0.706–5.820	0.189			
Hypertension	2.290	0.804–6.527	0.121			
Smoking	2.374	0.948–5.944	0.065			

**TABLE 3:** Multivariable analysis of risk factors of poor prognosis and measurement of the risk score.

	Multivariate analysis				
Variable	OR	95%CI	*P*-value	B coefficient	Points
PNI < 45.88	3.175	1.929–4.751	<0.001	4.497	4
MTD > 2.24 cm	1.106	1.026–2.431	0.002	2.241	2
Jaundice	2.961	1.037–4.931	0.037	2.403	2

**FIGURE 2:**
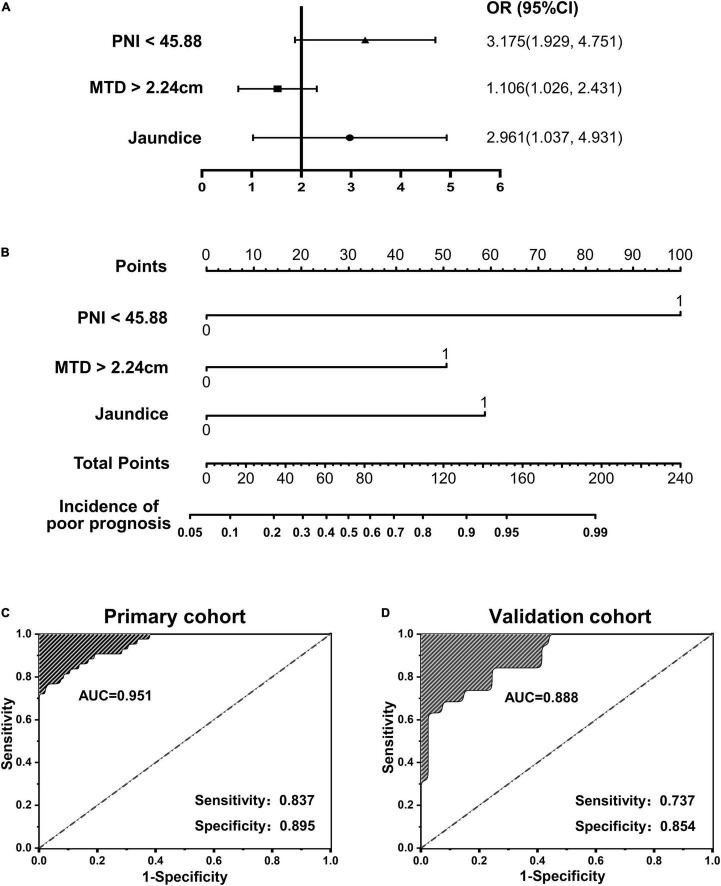
**(A,B)** Forest plot and nomogram plot of independent predictors of poor prognosis with odds-ratio (*OR*) multivariate regression model; **(C,D)** area under the curve (AUC) in the primary cohort and validation cohort.

To further verify the validity of this model, a forest plot of independent predictors of poor prognosis with the *OR* and a nomogram plot for predicting poor prognosis risk were presented in Figures 2A,B. It can be seen that PNI shows a higher score in predicting the incidence of poor prognosis, followed by MTD and JD. The AUC of the prediction model in the primary cohort was 0.951 (Figure 2C), and was 0.888 in the validation cohort (Figure 2D). To distinguish the incidence of poor prognosis in the high-risk group and the low-risk group for all study patients, according to the best Youden index of 0.610, we obtained an optimal cutoff value of 3.0.

**FIGURE 3:**
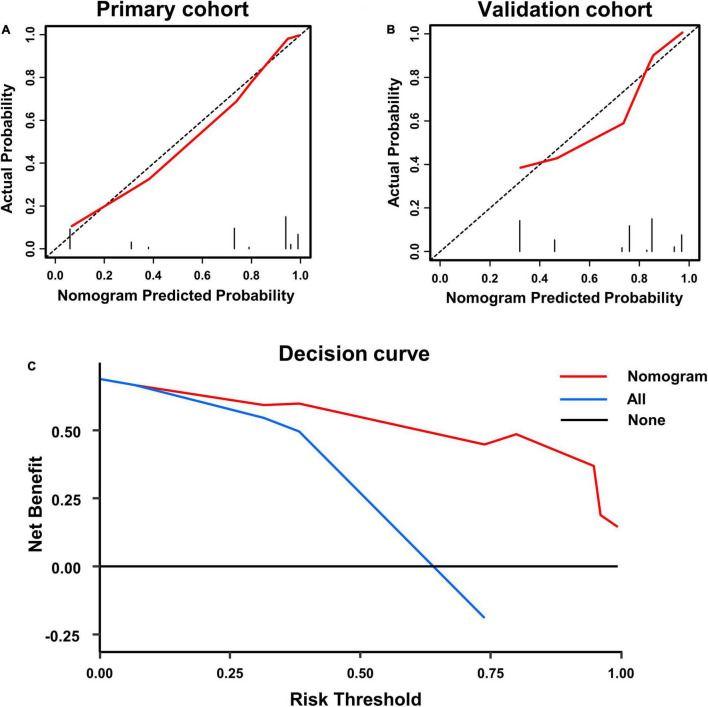
Calibration curve of poor prognosis prediction model in primary cohort **(A)** and validation cohort **(B)**; and **(C)** presents the decision curve for all patients.

A calibration analysis of this poor prognosis prediction model was presented in Figures 3A,B. The calibration curve and the lack of statistical significance in the H-L test (*p* was 0.090 in the primary cohort and was 0.192 in the validation cohort) indicate a reliable calibration. The decision curve shown in Figure 3C indicates that if the threshold probability is within a range from 0.05 to 0.99, the use of the nomogram can bring more net benefit to the patient than complete intervention or no intervention at all.

### Overall Survival and Recurrence

**FIGURE 4:**
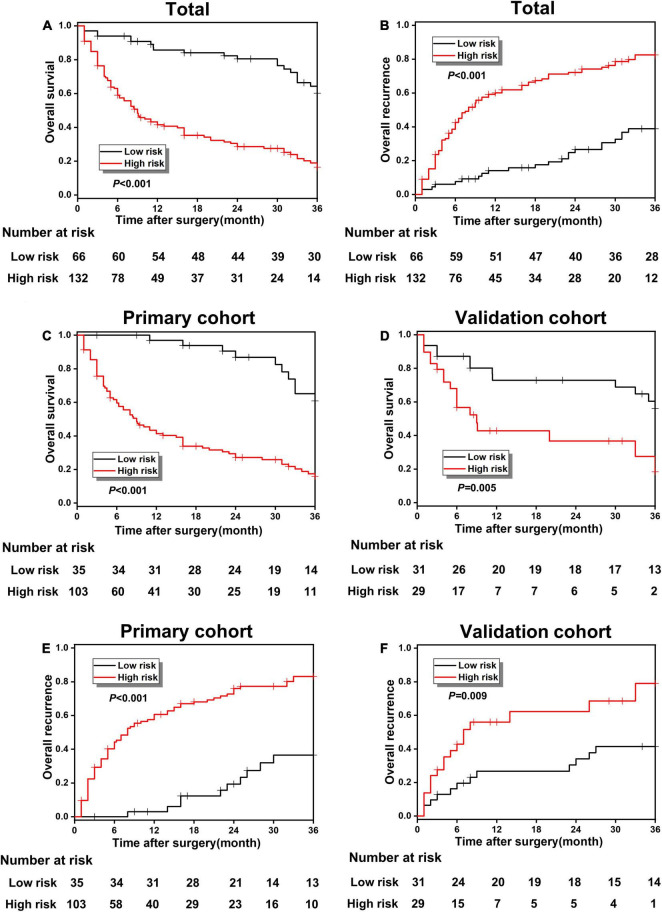
**(A,B)** The overall survival (OS) and recurrence comparison in the whole cohort between poor prognosis low-risk and high-risk groups; **(B–F)** the OS and recurrence comparison in the primary and validation cohort between the low-risk and high-risk groups.

According to the optimal cut-off value of 3.0, all study patients were divided into two groups with different risks of poor prognosis, including the low-risk group and high-risk group, and the KM survival analysis was carried out between the two groups to further effectiveness of risk classification based on the prediction model. The OS rate was 60.17% in the low-risk group and 16.43% in the high-risk group, which shows a significant statistical difference (*p* < 0.001; Figure 4A). The overall recurrence rate in the low-risk and the high-risk groups were 38.83 and 82.51%, respectively, indicating a significant statistical difference (*p* < 0.001; Figure 4B). Figures 4C,D has shown a great difference of the OS rate between low-risk and high-risk groups in the primary cohort (60.81 vs. 15.96%, *p* < 0.001) and validation cohort (56.12 vs. 18.34%, *p* = 0.005). The overall recurrence rate between the low-risk and high-risk groups in the primary cohort was 36.50 and 83.19%, respectively, and the difference was statistically significant (*p* < 0.001; Figure 4E). In the validation cohort, the overall recurrence rate between the low-risk and high-risk groups also showed a statistical difference (41.41 vs. 79.06%, *p* = 0.009; Figure 4F).

**FIGURE 5:**
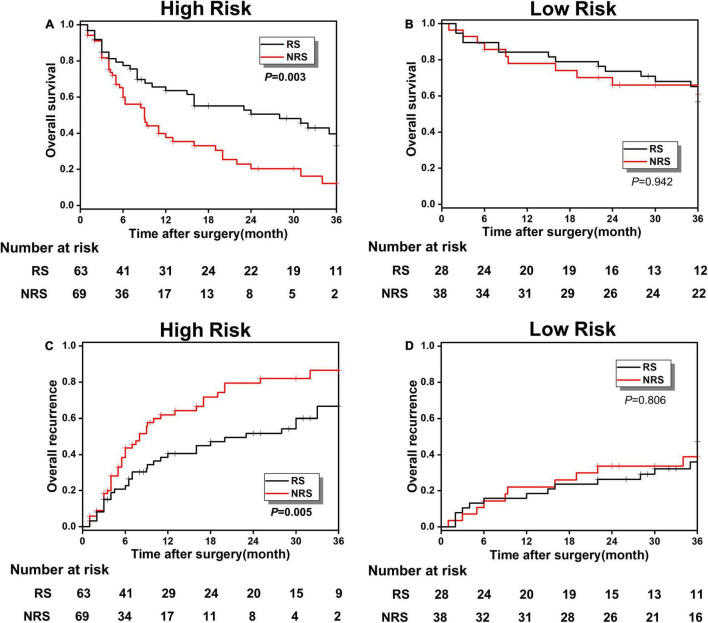
**(A,B)** The OS comparison between radical surgery (RS) and non-radical surgery (NRS) subgroups in the low-risk and high-risk groups; **(C,D)** the overall recurrence comparison between the RS and NRS subgroups in the low-risk and high-risk groups.

According to different surgical methods, such as RS (stands for radical resection) and NRS (stands for simple cholecystectomy), the patients in the poor prognosis low-risk and high-risk groups were classified into RS and NRS subgroups, respectively, and the KM survival analysis was conducted between RS and NRS subgroups. In the high-risk group, the OS rate in the RS and NRS subgroup were, respectively, 32.99 and 12.21%, indicating a significant statistical difference (*p* < 0.001; Figure 5A). Meanwhile, the overall recurrence rate between the RS and NRS subgroups also showed statistical difference (66.67 vs. 86.49%, *p* = 0.005; Figure 5C). While in the low-risk group, the OS rate and recurrence rate between RS and NRS subgroup indicated no statistical difference (*p* > 0.001; Figures 5B,D).

**FIGURE 6:**
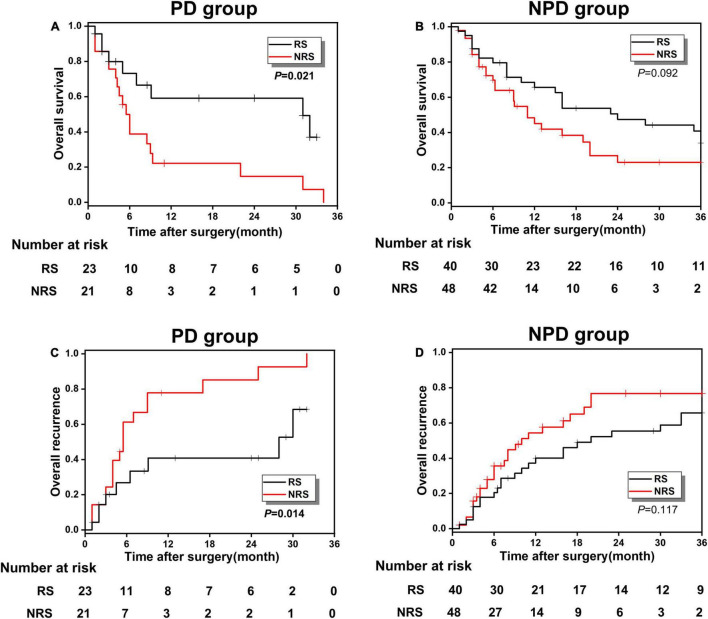
**(A,B)** The OS comparison between the RS and NRS subgroups in PD and NPD groups; **(C,D)** the overall recurrence comparison between the RS and NRS subgroups in PD and NPD groups.

As presented in Table 4, after univariable and multivariate regression analyses of the factors for predicting overall 3-years survival of study patients, we detected another risk factor, histological grade, in addition to PNI. Accordingly, its pathological grade could be categorized into PD and NPD (mainly including high and moderate differentiation). To further verify the survival relationship between histological grade and surgical methods, to provide important surgical guidance for the decision-making of patients with GBC, patients in the high-risk group were reclassified into the PD and NPD groups, and the KM survival analysis was conducted between RS and NRS subgroups. Figures 6A,C presented the OS and recurrence rate between RS and NRS subgroups in the PD group, which has shown statistical difference (*p* < 0.05). However, no statistical difference existed between RS and NRS subgroups in the NPD group (*p* > 0.05; Figures 6B,D).

**TABLE 4:** Univariable and multivariate regression analyses of factors for predicting overall 3-years survival of study patients.

	Univariate analysis			Multivariate analysis		
Variable	HR	95%CI	*P*-value	HR	95%CI	*P*-value
PNI < 45.88	0.258	0.168–0.396	<0.001	0.269	0.170–0.427	<0.001
MTD > 2.24 cm	0.731	0.513–1.043	0.084			
Gender	1.042	0.707–1.537	0.835			
Age < 60	0.985	0.725–1.539	0.458			
LN metastasis	0.661	0.465–0.941	0.025	0.859	0.556–1.327	0.493
TNM staging	0.449	0.243–0.832	0.011	0.769	0.324–1.822	0.551
CEA < 5 ng/mL	0.689	0.358–1.036	0.582			
CA199 < 40 kU/L	1.052	0.657–1.369	0.268			
PD	2.133	1.515–3.003	0.006	1.731	1.183–2.534	0.005
Pathology	0.958	0.503–1.825	0.896			
Jaundice	0.585	0.400–0.857	0.008	0.784	0.510–1.203	0.265
Cholelithiasis	1.212	0.862–1.704	0.270			
Tumor location	0.803	0.573–1.126	0.204			
Liver invasion	1.139	0.793–1.635	0.482			
Choledoch invasion	0.814	0.581–1.139	0.230			
Diabetes	0.852	0.578–1.256	0.419			
Hypertension	0.974	0.646–1.468	0.900			
Smoking	0.816	0.544–1.224	0.326			

## Discussion

In this study, a poor prognosis prediction model was established and validated by the hematological index, imageological data, and jaundice. With an AUC of 0.951 in the primary cohort and 0.888 in the validation cohort, this model contains PNI, MTD, and JD which demonstrates superior practicability and availability. Moreover, RS is beneficial to the long-term survival of patients with a high-risk of poor prognosis. For the patients with a low-risk of poor prognosis, a single cholecystectomy has little effect on the long-term prognosis. Nevertheless, in the high-risk group, patients with PD, RS is necessary, while for patients with high and moderate differentiation, RS has little effect on the long-term prognosis. Hence, a simple cholecystectomy is suitable to the GBC patients with high and moderate differentiation in the high-risk group instead of radical resection with great trauma. The significant findings provide a new therapeutic strategy for the clinical treatment of patients with GBC.

Low PNI was initially found to be an important predictor of a high risk of short-term postoperative complications in the gastrointestinal tract (24). The PNI can reflect the pretreated host immunological and nutritional status and thus affect postoperative survival. Recently, increasing evidence suggested that PNI was also related to OS in various types of malignancies, such as esophageal cancer and breast cancer (25–27). Our study demonstrated that the GBC patients with PNI < 45.88 were associated with a poor prognosis (AUC = 0.947; sensitivity, 0.767; and specificity, 0.958). In previous studies, the maximum tumor diameter (MTD more than 5 mm) has also been identified as a very important risk factor for poor prognosis for patients with primary hepatic carcinoma (28). The most likely reason is that the larger MTD is usually associated with capsular invasion, non-invasive growth patterns, satellite nodules, or tumor thrombi (29–31). Moreover, larger tumor size stimulates invasive behavior. Our study indicated that the GBC patients with MTD > 2.24 cm were related to a poor prognosis (AUC = 0.819; sensitivity, 0.842; and specificity, 0.767). In addition, some studies have confirmed that jaundice was a risk factor for the poor prognosis of cholangiocarcinoma and pancreatic ductal adenocarcinoma (32, 33). Patients with jaundice have cholestasis, usually associated with biliary tract infection, and poor surgical tolerance, which was consistent with our findings.

An early prediction of poor prognosis can effectively benefit preoperative or intraoperative individualized surgical plans, which have been verified by some experienced scholars (34–36). Ethun et al. analyzed 262 cases of accidental GBC from multiple centers and added the parameters, such as T stage, degree of differentiation, vascular invasion, and perineural invasion to establish the prediction model of local residual lesions, distant metastasis, and long-term survival (37). However, the evaluation of accidental GBC may be influenced by subjective factors and may have certain limitations. Mochizuki et al. established a risk scoring model for GBC by using the above four indicators (2–3 points for the low-risk group and 6–8 points for the high-risk group), and the scoring results were highly correlated with prognosis (38). This model has certain practicability, but lacks systematic evaluation and external verification, so the accuracy of the model has certain deficiencies. Bai et al. analyzed the data of 142 patients undergoing RS of GBC in Peking Union Medical College Hospital, which found that CA199, jaundice, tumor stage, and resection margin were independent prognostic factors through Cox regression model analysis (39). Then, they established the corresponding nomogram and evaluated the model accuracy through the subject operating characteristic curve and found that the prediction accuracy was good. However, the prediction effect of this model is not the optimal type (0.797–0.803). Established by the hematological, imageological indexes, and clinical manifestation, our prediction model demonstrated good predictive ability, which presented a higher prediction accuracy than single hematology index prediction models or radiomics. Furthermore, the AUC of this model in the primary cohort was 0.951, and in the validation cohort was 0.888, which indicates strong predictive performance.

The occurrence of poor prognosis will result in increased early recurrence rates. In this study, according to the best Yoden index, the patients were divided into a high-risk group and a low-risk group based on an optimal cut-off value. The 3-year survival of the high-risk group was lower than those of the lower-risk group in the primary and validation cohort. Meanwhile, our study found that RS could availably increase the long-term outcome of the high-risk group, which indicates that RS can effectively improve the postoperative OS of patients with GBC and reduce postoperative overall recurrence. Furthermore, the patients with RS or a simple cholecystectomy did not show a significant difference in OS and recurrence in the low-risk group. However, it does not mean that these patients can undergo simple cholecystectomy without RS in clinical practice. Studies have proved that simple cholecystectomy for stage T1b GBC had similar effects to RS, and there was no statistical difference in 5-year and 10-year postoperative survival rates (40, 41). Wang et al. suggested that a simple cholecystectomy was suitable for the GBC patients with T1b stage (AJCC 8th) and MTD < 1 cm (42). Therefore, preoperative comprehensive consideration should be taken from many aspects, such as TNM stage and tumor differentiation.

In addition, our study discovered that in the high-risk group, there was no significant difference of 3-year survival and recurrence in GBC patients with high and moderate differentiation, whether they underwent RS or a simple cholecystectomy. While patients with PD could obtain a long-term survival without recurrence after RS. Hence, for patients with PD and a high-risk score, when preoperative or intraoperative diagnosis of GBC is made, RS is highly recommended. Nonetheless, if the patient is postoperative diagnosed of accidental GBC, due to the few significant difference of 3-year survival and recurrence for the patients with high and moderate differentiation or a low-risk prediction score, rather than a traumatic RS, a simple cholecystectomy can be considered for acceptance.

However, this study has certain limitations. First of all, a single-center retrospective study may not have such a high level of evidence, and the results are not strongly persuasive. Second, the data included in this study are insufficient (only 198 patients), so there may be some deviations in the results. In addition, our prediction model is established by PNI, MTD, and jaundice, but other clinical characteristic parameters, such as tumor margins, invasion, and metastasis, have not been comprehensively evaluated. Hence, the issues mentioned above need to be further verified by more and larger participants, multicenter randomized controlled studies, and this is also the research plan that we need to carry out further in the future.

## Conclusion

In summary, we have developed and validated a novel poor prognosis prediction model based on PNI, MTD, and jaundice for patients with GBC, which shows superior practicability and availability. Due to a high-risk score of early tumor recurrence, our findings demonstrate that RS is necessary for those preoperative or intraoperative diagnosis of patients with GBC. Nevertheless, for those postoperative accidental diagnosis of GBC, whereas for patients with PD and a high-risk score, RS is highly recommended; while for the patients with high and moderate differentiation or a low-risk score, rather than a traumatic RS, a simple cholecystectomy can be considered for acceptance. These findings demonstrate important guiding significance for the next treatment strategy of accidental GBC which occasionally appears in clinic.

## Data Availability Statement

The original contributions presented in the study are included in the article/supplementary material, further inquiries can be directed to the corresponding authors.

## Ethics Statement

The studies involving human participants were reviewed and approved by the Ethics Committee of the Medical Faculty of Fujian Medical University. The patients/participants provided their written informed consent to participate in this study.

## Author Contributions

SQ and PC contributed to the conception of the study. HH, ZY, and GC contributed to the data collection and audit. SQ and PC performed the data analyses and wrote the manuscript. All authors contributed to the article and approved the submitted version.

## Conflict of Interest

The authors declare that the research was conducted in the absence of any commercial or financial relationships that could be construed as a potential conflict of interest.

## Publisher’s Note

All claims expressed in this article are solely those of the authors and do not necessarily represent those of their affiliated organizations, or those of the publisher, the editors and the reviewers. Any product that may be evaluated in this article, or claim that may be made by its manufacturer, is not guaranteed or endorsed by the publisher.

## Abbreviations

PNI: prognostic nutrition index
MTD: maximum tumor diameter
LNM: lymph node metastasis
NRS: non-radical surgery
RS: radical surgery
JD: jaundice
PD: poorly differentiation
NPD: non-poorly differentiation.
